# 

**Published:** 1937

**Authors:** 


### The Medical Library of the University of Bristol,

WITH WHICH IS INCORPORATED

**The Library of the Bristol Medico-Chirurgical Society.**

---

*The following donations have been received since the publication of the list in July,* 1937.

September, 1937.

| | | |
|---|---|---|
| Rear-Admiral C. S. Butler (1) .. .. .. | 1 | volume. |
| Dr. J. D. A. Gray (2) .. .. .. .. | 1 | ,, |
| Professor E. W. Hey Groves (3) .. .. | 7 | volumes. |
| Michell Clarke Fund (4) .. .. .. | 1 | volume. |
| University of Buenos Aires (5) .. .. | 1 | ,, |
| Miss D. Anne Norburn (6) .. .. .. | 1 | ,, |
| Dr. P. Watson-Williams (7) .. .. .. | 2 | volumes. |

Unbound periodicals have also been received from Professor E. W. Hey Groves, Professor C. Bruce Perry, Dr. J. Odery Symes, and Mr. P. Watson-Williams.

### THE ONE HUNDRED AND SIXTIETH LIST OF BOOKS.

The figures in round brackets refer to the figures after the names of the donors. The books to which no such figures have been attached have either been bought from the Library Fund or received through the *Journal.*

**Aitken, William** .. *Handbook of Treatment* .. .. .. .. .. (7) 1887

**Barilari, Mariano J.**.. *Hechos clinicos y su interpretación* .. .. 1937

**Bertwistle, A. P.** .. *The Rôle of Chemiotaxis in Bone Growth* (3) 1937

**Browne, F. J.** .. .. *Obstetric Technique* .. .. .. .. .. .. .. .. 1937

**Browne, G. L., and Stewart, C. G.** *Reports of Trials for Murder by Poisoning* .. .. .. .. .. .. .. .. (2) 1883

| | | | |
|---|---|---|---|
| **Butler, Charles S.** | *Syphilis sive morbus humanus* | (1) | 1936 |
| **Cancer Control Organization.** | *Cancer*, Edinburgh, 1937 | (3) | 1937 |
| **Conybeare, J. J.** [Ed.] | *Textbook of Medicine* | 3rd Ed. | 1936 |
| **Dioscorides** | *Works* | (6) | 1478 |
| **Dupré, A., and Wilson, H.** | *Short Manual of Inorganic Chemistry* | 2nd Ed. Rev. (7) | 1892 |
| **Ernst, Jørgen** | *Hautesinfektionsprobleme* | (3) | 1937 |
| **Garcia, Carlos L.** | *Equinococosis hidatidica pulmonar en la infancia* | (5) | 1929 |
| **Grüneberg, Hans** | *Elementary Genetics* | | 1937 |
| **Hall, I. Simon** | *Diseases of the Nose, Throat and Ear* | | 1937 |
| **Harvey, W. C., and Hill, H.** | *Milk Products* | | 1937 |
| **Hill, Charles, and Clegg, H. A.** | *What is Osteopathy?* | | 1937 |
| **Hutchison, Robert** | *The Elements of Medical Treatment* | 3rd Ed. | 1937 |
| **Kulenkampff, D.** | *Allgemeine Chirurgie* | 7th Ed. Rev. (3) | 1937 |
| **Labey, G., and Leveuf, J.** | *Chirurgie du membre inférieure* | 5th Ed. (3) | 1923 |
| **Lauwers, E. E.** | *Introduction à la chirurgie* (4 parts) | (3) | 1932–6 |
| **Lees, David** | *Practical Methods in the Diagnosis and Treatment of Venereal Diseases* | 3rd Ed. | 1937 |
| **Rea, Marguerite Requa** | *The Diet Book* | 3rd Ed. | 1937 |
| **Ross, T. A.** | *The Common Neuroses* | 2nd Ed. | 1937 |
| **Roxburgh, A. C.** | *Common Skin Diseases* | 4th Ed. | 1937 |
| **Ryle, John A.** | *The Natural History of Disease* | | 1937 |
| **Scott, G. Laughton** | *The Morphine Habit* | 2nd Ed. | 1937 |
| **Sewart, Dorothy** | *The Medical Cookery Book* | | 1935 |
| **Shields, Clive** | *Hay Fever* | | 1937 |
| **Unti, Ovidio** | *Anestesia de base pela Dialilmaloniluréa* | (3) | 1936 |
| **Wolff, Eugene** | *Diseases of the Eye* | | 1937 |

### TRANSACTIONS, REPORTS, JOURNALS, ETC.

| | | | |
|---|---|---|---|
| Collected Papers of the Mayo Clinic. | Vol. XXVIII | (4) | 1936 |
| Hospitals Yearbook, 1937 | | | 1937 |
| Surgical Clinics of North America. | Vol. 17, No. 3 (June, 1937) | | 1937 |

### Publications Received

From Baillière, Tindall & Cox. :

*Modern Treatment in General Practice.* Vol. III. Edited by Cecil P. G. Wakeley, D.Sc., F.R.C.S., F.R.S.E. Price 10s. 6d.

*Minor Maladies and their Treatment.* By Leonard Williams, M.D. 7th Edition. Price 10s. 6d.

From H. K. Lewis & Co. Ltd. :

*Materia Medica for Nurses.* By A. Muir Crawford, M.D., F.R.F.P.S. 4th Edition. Price 3s. 6d.

*Reports on Chronic Rheumatic Diseases.* No. 3. Price 10s. 6d.

From E. & S. Livingstone :

*Medico-legal Aspects of the Ruxton Case.* By John Glaister, M.D., D.Sc., and James Couper Brash, M.A., M.D., F.R.C.S. Ed. Price 21s.

From Macmillan & Co. Ltd. :

*Diseases of the Heart.* By Sir Thomas Lewis, C.B.E., F.R.S., M.D., D.Sc., LL.D., F.R.C.P. 2nd Edition. Price 12s. 6d.

From Medical Publications Ltd. :

*Post-Graduate Surgery.* Vol. III. Edited by Rodney Maingot, F.R.C.S., Price 70s.

From Oxford University Press :

*A Criticism of Nursing Education.* By Harold Balme, M.D., F.R.C.S. D.P.H. Price 2s. 6d.

*The Abdominal Surgery of Children.* By Sir Lancelot Barrington-Ward, K.C.V.O., Ch.M., F.R.C.S. 2nd Edition. Price 25s.

*The Scientific Basis of Physical Education.* By F. W. W. Griffin, M.A., M.D., B.Ch. Price 7s. 6d.

*Skin Diseases in General Practice.* By H. Haldin-Davis, D.M., M.A., F.R.C.P. 3rd Edition. Price 17s. 6d.

*A Text-book of the Practice of Medicine.* By Frederic W. Price (Ed.), M.D., C.M., F.R.C.P., F.R.S. 5th Edition. Price 36s. Also India Paper Edition. Price 45s.

*The Diet Book.* By Marguerite Requa Rea. 3rd Edition. Price 6s.

*Infants in Health and Sickness.* By Robert Elsworth Steen, M.D., F.R.C.P.I. Price 5s.

From SWEDEN ROYAL MEDICAL BOARD :

*Investigations into Questions of Social Hygiene in the Counties of Västerbotten and Norrbotten, Sweden.*

From JOHN WRIGHT & SONS LTD. :

*Synopsis of Obstetrics and Gynæcology.* By ALECK W. BOURNE, M.A., M.B., B.Ch., F.R.C.S., F.C.O.G. 7th Edition. Price 15s.

*British Journal of Surgery.* Vol. XXV. No. 97. Price 12s. 6d.

*The Elements of Medical Treatment.* By ROBERT HUTCHISON, M.B., LL.D., D.Sc., F.R.C.P. 3rd Edition. Price 5s.

*Treatment of some Chronic and Incurable Diseases.* By A. T. TODD, O.B.E., M.B., M.R.C.P. Price 10s.

### Local Medical Notes

EXAMINATION RESULTS.

**University of Bristol.**—Students of the University have recently passed the following examinations :—

M.B., Ch.B.—*Supplementary First Examination. Pass* : J. H. Challenger, Margaret J. Griffith, Eileen M. Herbert, J. C. O. Iwenofu, A. R. B. McGillycuddy, A. J. F. Saxton, W. H. N. Scawin, W. H. E. Turnbull, Jean Wallace.

B.D.S.—*Supplementary First Examination. Pass :* R. M. Sharp.

L.D.S.—*Supplementary First Examination. Pass :* V. T. Scard.

**Conjoint Board.** — M.R.C.S., L.R.C.P. *Pass : In Anatomy :* B. C. E. Aldous. *In Pharmacology and Materia Medica :* Mary R. More. *In Medicine and Surgery :* A. M. Spencer.* *In Pathology, Medicine and Midwifery :* K. G.

* Qualified.

Bergin. *In Midwifery :* R. L. J. S. Derham, Rotha H. Dalton, Mabel L. Glenny, Ruth Jones. *In Pathology and Midwifery :* Myrtle M. Hutchins. *In Pathology :* P. W. M. Martin, Dorothy E. Dickinson, Margaret E. Morgan, Margaret E. M. Slater, R. D. Cæsar. *In Surgery :* Ursula W. Wood.

---

### APPOINTMENT.

A. W. Falconer, C.B.E., D.S.O., M.D. (Aberdeen), M.R.C.P. (Lond.), Professor of Medicine at Cape Town University, has been appointed Vice-Chancellor of that University. Professor Falconer was House Physician at the Bristol Royal Infirmary in 1907.

